# Tracking progress towards equitable child survival in a Nicaraguan community: neonatal mortality challenges to meet the MDG 4

**DOI:** 10.1186/1471-2458-11-455

**Published:** 2011-06-09

**Authors:** Wilton Pérez, Rodolfo Peña, Lars-Åke Persson, Carina Källestål

**Affiliations:** 1 Health and Demographic Research Centre, CIDS, National Autonomous University, León; Nicaragua (UNAN; 2 International Maternal and Child Health (IMCH), Department of Women's and Children's Health, Uppsala University, Sweden

## Abstract

**Background:**

Nicaragua has made progress in the reduction of the under-five mortality since 1980s. Data for the national trends indicate that this poor Central American country is on track to reach the Millennium Development Goal-4 by 2015. Despite this progress, neonatal mortality has not showed same progress. The aim of this study is to analyse trends and social differentials in neonatal and under-five mortality in a Nicaraguan community from 1970 to 2005.

**Methods:**

Two linked community-based reproductive surveys in 1993 and 2002 followed by a health and demographic surveillance system providing information on all births and child deaths in urban and rural areas of León municipality, Nicaragua. A total of 49 972 live births were registered.

**Results:**

A rapid reduction in under-five mortality was observed during the late 1970s (from 103 deaths/1000 live births) and the 1980s, followed by a gradual decline to the level of 23 deaths/1000 live births in 2005. This community is on track for the Millennium Development Goal 4 for improved child survival. However, neonatal mortality increased lately in spite of a good coverage of skilled assistance at delivery. After some years in the 1990s with a very small gap in neonatal survival between children of mothers of different educational levels this divide is increasing.

**Conclusions:**

After the reduction of high under-five mortality that coincided with improved equity in survival in this Nicaraguan community, the current challenge is the neonatal mortality where questions of an equitable perinatal care of good quality must be addressed.

## Background

The world has achieved an impressive progress of child survival during the last few decades. The number of deaths in children aged less than five has declined from thirteen million by 1990 to nine million in 2007 - nevertheless this progress is insufficient [1]. Of the eight Millennium Development Goals (MDGs) that the world should meet by 2015, the MDG-4 calls for a two-third reduction of under-five mortality. Only 34% percent of countries in the world are on track to meet this goal [2]. In spite of the worldwide progress in child survival, the *Countdown *to 2015 reported that out of 68 priority countries, most of them in Sub-Saharan Africa and South Asia, have shown no progress (17 countries) and insufficient progress (32 countries) to meet the MDG-4 [3].

A major part of under-five mortality occurs in the neonatal period [1]. Every year around four million newborns die during the first 28 days of life, three quarters of these deaths occur in the first week and 99 percent take place in developing countries [4]. Child health programs and interventions have usually been effective in preventing deaths after the first month of life. In order to reach the MDG-4 priority must be given to the mother-and -newborn dyad. A small number of affordable and evidence-based services and interventions could result in a substantial reduction of neonatal mortality [5].

Globally child survival chances are characterised by huge and unfair differentials between countries and regions. In 2007 the under-five mortality rate (U5MR) in developing compared to developed countries was 74 and 6 per thousand live births, respectively [6]. This inequity is also seen within countries, where family economy and other social characteristics are linked to the child's survival chances. There is a tendency that health interventions primarily reach children of the wealthier families and much later benefit the poorer segments of society [7,8]. It is estimated that if selected low cost evidence-based newborn interventions could achieve a high coverage (> 90%) almost three million of neonatal deaths could be prevented [9], and in Asia and Africa 33-66% of newborn lives would be saved [10].

To monitor the MDG-4, data sources of good quality are needed [11,12]. In developing countries, vital statistics are of poor validity to generate reliable figures for monitoring continually health indicators. Most recently, household surveys that record the full birth history of the mother have been used in national and local studies on child health and mortality in countries of poor data sources. All births and child deaths are registered and used to estimate the child mortality. Other relevant information to analyze determinants is also collected. With the lack of good statistics, that new methodologies have been useful for monitoring, evaluating and planning strategies that help to clarify priorities in the reduction of child mortality and reducing inequities [13].

### Child survival in Nicaragua

Nicaragua is one of the countries in Latin America where child survival has improved rapidly over the past few decades. Our group has previously reported a decline in infant mortality in León, Nicaragua, from the level of 120 to 40 infant deaths per 1 000 live births from the late 1960s to the beginning of the 1990s [14]. This improvement was seen during a time of severe political, economic and social turmoil that followed the revolution in 1979. Progress in child survival was most likely a result of pro-poor targeted and decentralized public health services, giving priority to mothers and children [15].

Nicaragua seems to be in progress towards the MDG-4 [6]. The U5MR declined from 65 to 30 per 1000 live births between 1990 and 2009 [16]. Even if Nicaragua is on track towards the MDG4 based on the overall level of U5MR, barriers as inequalities and newborn health could desacelerate this progress. The U5MR is higher in rural (55 per 1000) in comparison to urban (34 per 1000) areas; and among the poorest group the U5MR is 3.3 times higher than among the wealthiest group. On the other hand neonatal mortality has only shown a minor decline (from 20 to 16 deaths per 1 000 live births) from 1993 to 2006 [16]. Thus, this stagnation contrasts to the earlier rapid reduction in post-neonatal and child mortality.

During the late 1990s and early 2000s the Nicaraguan health care system underwent major change, resulting in an increase in private health services, also within reproductive and child health [15]. The country has a problematic economic situation with an estimated unemployment of 46% in 2005 [17].

This study aims at analysing trends and social differentials in neonatal and under-five mortality in the municipality of León, Nicaragua, and to relate the findings to the forth Millennium Development Goal as well as to a health equity perspective.

## Methods

### The setting and research design

The study was carried out in León municipality, a city located in the Pacific part of Nicaragua 93 km from the capital. It has a population of 174 051 inhabitants (2005), where 80% live in urban areas, and 46% of the population is under 18 years of age [18]. León municipality is considered the second most important city in Nicaragua after the capital with a university hospital, which it is a referent hospital for the closest cities. According to the Human Development Report, in 2002 the human development index in Nicaragua ranked from 0.37 to 0.82 [19]. León municipality had a human development index of 0.74 a value shared for most cities located close to the capital. From 2002 and onwards a Health and Demographic Surveillance System (HDSS) has been running, updating demographic information every six to twelve months and providing prospective information on pregnancies, births and child survival until the end of the current study period December 31, 2005 [20]. This HDSS was based on a linkage on household and individual level to a cross sectional study data base. The data in this data base was collected from a 22% sample of the total population in León municipality selected in 1993, using a cluster-sampling technique described elsewhere [14]. The follow-up survey was performed in 2002, providing updated and linked information on demographic characteristics and selected health indicators. This 2002 HDSS data base included a total of 54 647 people residing in 10 994 households. Only eleven of the households (0.1%) declined to participate in the survey.

Female field workers performed the data collection and interviewed women of reproductive age in their homes. Quality control routines were realized during fieldwork and data entry.

Women of reproductive age (15-49) were interviewed with respect to their reproductive and birth histories including dates of births, outcome of each pregnancy and, when relevant, the date of the child's death. Data collection was supported by the use of a local events calendar. Data were also collected on educational level of the mother, age at delivery, parity, and domicile. However, for place and attendance at delivery data was collected only from the start of the surveillance (2003) and only for women resident in the study area.

Under-five mortality rate was defined as the number of deaths before five years of age per 1000 live births. The U5MR was disaggregated into neonatal (< 28 days), post neonatal (28 days-11.9 months) and child mortality (1-4.9 years). Mother's age and parity were calculated for the time of each childbirth. Parity was categorized into three levels; one, two to four, and more than four live births, respectively. Place of delivery was categorized as hospital, health centre, private clinic, home or other place. Attendant at delivery was classified into physician, nurse, midwife or other person. Women's educational levels were classified into no formal education (either illiterate or literate but had not completed primary school) or formal education (completed primary school or higher level of education). Domicile of the mother was defined as urban or rural.

### Analyses

Place of and attendance at delivery during the last 3-year period were analysed and differences between rural and urban areas were tested (Chi^2^). Data on births, deaths and neonatal, post-neonatal and child mortality rates were analysed for 3-year periods from 1970 to 2005. Mortality curves were prepared with 3-year moving averages in order to smooth random fluctuations in the material. The U5MRs for rural and urban areas were graphically represented. Comparisons of rates or proportions were supported by Chi^2 ^tests. Relative risk of neonatal death (Hazards ratios with 95% confidence intervals) and relative risk of under-five death were analysed by Cox proportional hazard analyses, considering educational level of the mother during three different time periods from 1970 to 2005. Adjustments were done for parity and domicile. Data entry and managing were done using Microsoft Access 2000 (Microsoft Corporation, Washington), while analyses were performed in Statistical Package for Social Sciences (Version 14.0; SPSS Inc, Chicago, USA).

## Results

For the period 1970-2005, a total of 17 673 mothers had 49 972 live births and 2 653 under-five deaths were recorded (1191 neonatal deaths, 1044 post-neonatal and 418 child deaths).

During 2003-2005, the proportion of deliveries taking place within the health system (hospital, health centre, and private clinic) was 96.8%. This figure was higher in urban (99.5%) than in the rural (89.5%) areas (p < 0.01), table 1. Consequently, skilled personnel attended most deliveries. In the urban domicile physicians attended almost all deliveries (99.1%) - less so in the rural area (87.7%, p < 0.01).

**Table 1:** Place and attendance at deliveries in rural and urban Leon municipality, Nicaragua, 2003-2005

	Total n = 2090 (%)	Rural n = 564 (%)	Urban n = 1,525 (%)
**Place of delivery**			
Hospital	82.9	86.7	81.5
Health centre	0.2	0.5	0.1
Private clinic	13.7	2.3	17.9
Home	3.0	9.6	0.5
Other	0.2	0.9	0.0
			
**Attendant at delivery**			
Physician	96.1	87.8	99.1
Nurse	0.8	2.0	0.3
Midwife	2.6	8.5	0.4
Other	0.6	1.8	0.1

**Figure 1:**
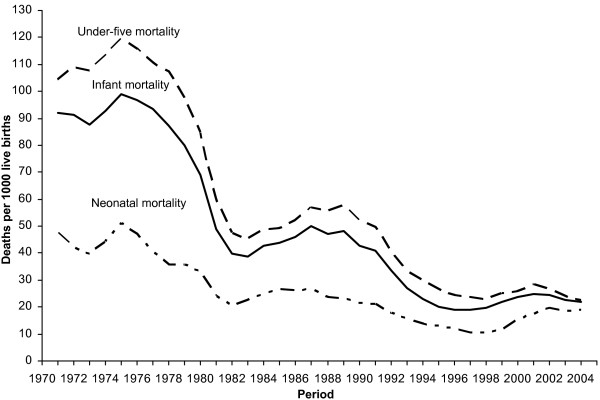
**Under-five, infant and neonatal mortality rates 1970-2005, León municipality, Nicaragua (3 years moving averages)**.

**Figure 2:**
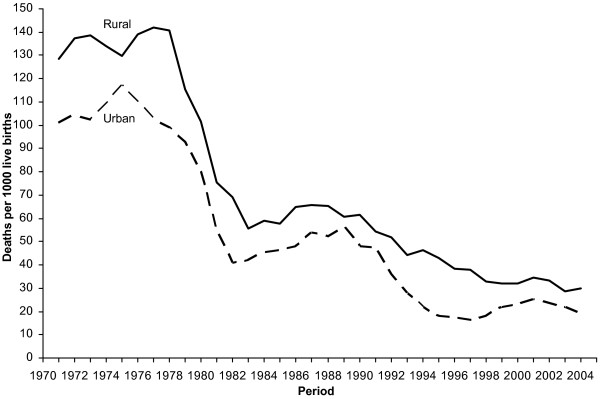
**Under-five mortality rate in rural and urban areas 1970-2005, León municipality, Nicaragua, (3 years moving averages)**.

Under-five survival increased considerably in León from 1970 to 2005. The U5MR dropped from the level of 103/1000 live births in the early 1970s to 23/1000 in 2003-2005 (table 2 and figure 1). The reduction in U5MR was rapid during the late 1970s and 1980s, with a very sharp decline especially in the rural areas (figure 2).

**Table 2:** Neonatal, post-neonatal and child mortality rates 1970-2005, León municipality, Nicaragua

Period	Live births	Neonatal mortality (< 28 d)		Post-neonatal mortality (28 d -11.9 m)		Child mortality (1-4.9 y)	
		
		Deaths	Rate*	Deaths	Rate*	Deaths	Rate*
1970-72	1605	74	46.1	70	43.6	21	13.1
1973-75	2425	108	44.5	116	47.8	50	20.6
1976-78	3142	123	39.1	167	53.2	53	16.9
1979-81	4405	145	32.9	156	35.4	69	15.7
1982-84	4873	111	22.8	78	16.0	31	6.4
1985-87	5235	137	26.2	103	19.7	34	6.5
1988-90	6039	141	23.3	148	24.5	57	9.4
1991-93	6406	115	18.0	99	15.5	45	7.0
1994-96	3982	52	13.1	28	7.0	27	6.8
1997-99	3987	43	10.8	36	9.0	13	3.3
2000-02	4326	75	17.3	33	7.6	15	3.5
2003-05	3547	67	18.9	11	3.1	2	0.6

* Rate: Deaths per 1 000 live births

Neonatal mortality rate (NMR) decreased from 46 to 19/1000 live births, implying that neonatal deaths constituted 45% of under-five deaths in the early 1970s and 83% of the under-five deaths in 2003-2005. In the mid-1990s the neonatal mortality was remarkably low (10.8/1000) followed by a gradual increase, maybe most visible in the urban area (figure 2). The neonatal mortality rate 1994-1999 was significantly lower in comparison with the later period 2000-2005 (p = 0.01, Table 2).

The proportion of women of reproductive age with formal education in the surveys in 1993 and 2002 was 37% and 72%, respectively. The gap in neonatal mortality between educational groups (no formal education vs. formal education) varied over time. In the first third of the study period, 1970-1981, the gap was pronounced in neonatal and under-five mortality between educational levels of the mothers (table 3). In the mid-period (1982-1993) the gap was very small (NMR 23.4 and 20.5/1000 live births, respectively), while the last period again showed a tendency towards larger differentials in neonatal mortality.

**Table 3:** Under-five mortality and neonatal mortality hazard risks (HR) in relation to educational level of the mother and time period.

Period	Education	Neonatal mortality		Under-five mortality	
		NMR	HR (95%CI)	U5MR	HR (95%CI)
1970-1981	No formal education	45.6	3.3(2.7, 4.0)	116.6	5.7(4.9-6.6)
	Formal education	26.9	2.0(1.6, 2.6)	71.2	3.4(2.9-4.0)
1982-1993	No formal education	23.4	1.6(1.3, 1.9)	56.8	2.5(2.1-2.9)
	Formal education	20.5	1.5(1.2, 1.8)	39.5	1.8(1.5-2.1)
1994-2005	No formal education	20.0	1.2(0.9, 1.7)	39.7	1.5(1.2-1.9)
	Formal education	13.5	1	21.0	1

Children of mothers with formal education level 1994-2005 is set as reference. Cox proportional hazards analyses adjusted for parity and domicile

## Discussion

We have shown that under-five mortality in this Nicaraguan community rapidly dropped during the 1980s, mainly linked to a pro-poor provision of essential lifesaving interventions to those most in need. Some coverage interventions as education increased reducing the illiterate rate 37% in rural compared to 25% in urban areas, health posts and centers providing preventive and curative services were built increasing the use by 7.5% in 1971 to 10.1% by 1980, the level of stunting was reduced from 36% in 1966 to 22% in 1986 [21]. Births in health institutions increased 73% between 1978-1989 and immunization increased. This community is on track to reach the Millennium Development Goal 4 of a two-thirds reduction of under-five mortality 1990-2015. However, there is an increasing divide in child survival between mothers with different education levels, and neonatal mortality is increasing in spite of a very high proportion of deliveries within the health system during 1994-2005.

### Rapid decline in mortality

We have shown a rapid decline in under-five mortality from the middle of 1970s to the early 1980s. During that turbulent period in Nicaragua the new government prioritized the under-served part of the population [22], and the health budget increased from 3% to 11% of the Gross Domestic Product (GDP). Child deaths caused by infectious diseases were prevented in part by an increased coverage of immunizations, oral rehydratation treatment, management of infectious diseases and sanitation improvements [21,22]. Emphasis was given to community participation, especially in rural regions and basic education and health promotion were implemented [22]. The war from 1983 to 1990 and the economic embargo imposed by the United States of America were some of the factors that led to a reduction of the health budget to 6.6% of the GDP in 1989 [23]. During that period under-five mortality increased, maybe as a result of lack of resources within the health system, lack of food, low economic resources in the family, high proportion of deliveries at home in those regions far from the health system, poor secondary preventions and tertiary preventions of child diseases and reduced access to clean water [24]. At the end of the war in 1990 there was a new political change, moving towards a decentralized health care system, with an increased private sector [15]. The under-five mortality decreased in spite of reduced public health expenditure. However, the potential benefits of the increasing private health sector services were only in urban areas, where 80% of the population is concentrated, the main university hospital is located and families of higher socioeconomic strata dominate.

### Social equity in child survival

Mother's education is frequently used as a social marker associated with child health and survival [25]. A wide gap in child survival between maternal education groups usually indicates a social variation in the uptake of preventive and curative services along the continuum of care for the pregnant woman and her offspring [26]. The remarkable reduction in mortality combined with a much smaller social gap in under-five and neonatal mortality that we have demonstrated in this Nicaraguan community (Table 3) suggests that the health system during the 1980s managed to scale up the essential services and to target those most in need [14]. This illustrates that improved child survival is not always associated with an increasing gap in mortality between social groups [27]. However, the last study period (1994-2005), the social divide in under-five and neonatal mortality has increased - in spite of being on track for the MGD-4. This reinforces the importance of adding an equity measure to the millennium development goal indicators [28]. The current health system is more complex with an increasing public-private mix in the available services for mothers and children. It is beyond the scope of this analysis to clearly identify the reasons to this increasing divide, but there are reasons to question whether mother and child health services are equitably distributed and maintain an adequate quality of care.

To live in rural settings is frequently described as a disadvantage for child health and survival [29]. However, in this Nicaraguan community big efforts were made during the 1980s to improve child health and survival in the rural areas and to reduce the rural-urban inequity. The government policy included community participation with community health workers called *brigadistas *to promote basic education and health promotion [30]. During the period of war 1983-1990 this community model was disintegrated and child mortality increased. When the war ended social programs and access to health services were re-established Private health services were only offered in urban areas, increasing their accessibility, although evidence of the quality of these services has not been assessed in connection to improve the child survival. However, a higher access to health services, better socio-economic status, good sanitation, access to clean water could have contributed to a more rapid reduction in under-five mortality in urban as compared to rural areas increasing the inequity in survival between the two areas. This pattern continued until 1997, when urban areas faced increasing under-five mortality. A rural-urban migration in part forced by the Hurricane Mitch in 1998 could explain this increase, and the mortality increase is most likely a reflection of the precarious conditions of the urban slums [31].

### Neonatal survival

Many countries face a major obstacle to reach the MDG-4 - the persistent neonatal mortality. In this Nicaraguan community neonatal mortality was reduced, but at a lower speed than the reduction in post-neonatal and child mortality rates - a pattern found in many other societies in transition [32]. A successful audit-and-feedback process of change in perinatal services was initiated at the regional hospital in the early 1990s in order to improve the quality of care provided. That process resulted in 85% reduction of early neonatal mortality in the hospital [33], this was also reflected in the community-based data reported in this paper where NMR declined 50% during 1985-1993 (period of hospital intervention).There is usually an association between access to and utilisation of a continuum of good quality maternal and neonatal health care and neonatal survival [5,9]. But efficacious services and interventions do not always reduce inequity in survival chances [9,13,27,28,34]. The recent development in neonatal mortality in this local Nicaraguan community raises questions of an equitable access to a continuum of perinatal services and about the quality of care that is provided. Further, these data illustrates the need for an equity stratification or adjustment in the MDG-4 monitoring and reporting of under-five mortality - otherwise, an increasing social divide in survival chances can be hidden in data showing a society on track to reach the child mortality goal.

### Methodological considerations

Our study linked two retrospective reproductive life surveys (1993 and 2002) to a continuous health and demographic surveillance that followed (from 2002). The recording of retrospective data was performed in accordance with well-known and used methods in this type of surveillance. To minimize the recall bias of dates of birth and death a local events calendar was used in the interviews. In the Nicaraguan setting there is no reason to believe that women purposively should misreport births and/or deaths. The refusal rate was very small (< 0.1%) that minimizes the risk of selection bias [14,20].The interviews with the included women were performed in privacy by trained female field workers in order to create confidence and reduce the risk of misreporting of childbirths and deaths.

Previous out-migration and maternal deaths could potentially distort our estimation of mortality in childhood during the past decades. However, it is unlikely that out-migration has had any major influence on trends and mortality differentials over time that are reported by the mothers who continue to live in the study area. This possible bias does not exist from 2002, when the surveillance system was initiated. Maternal mortality is - in spite of being a public health problem - not of the magnitude that influences aggregated child survival data.

Factors like educational level and rural/urban domicile were mainly used as stratifying variables when analyzing gaps between social groups. The mother's education was measured at time of interview as the number of years of schooling and this information was updated when the surveillance system was initiated.

The results shown in this study are not representative of Nicaragua as a whole. However, the municipality of León is relatively typical of the Pacific part of Nicaragua, and our findings are consistent with the corresponding results of national census and demographic surveys [16,18].

## Conclusions

The study concluded that this local community in Nicaragua is on track for reaching the MDG-4 by 2015. Despite of the substantial progress gained in child survival new challenges emerge from our data. To reduce the U5MR and achieve the MDG-4 by 2015 emphasis should be addressed to reduce the neonatal mortality. Finally, any kind of interventions addressed to improve the child survival must use an equity approach.

## Competing interests

The authors declare that they have no competing interests.

## Authors' contributions

WP participated in part of data collection, developed the statistical analysis, and drafted the manuscript. RP designed the study, supervised the full data collection, contributed with the discussion and revision of the manuscript. LAP and CK participated in the design of the study, data interpretation, revision and writing of the manuscript. All authors read and approved the final manuscript.

## Pre-publication history

The pre-publication history for this paper can be accessed here:

http://www.biomedcentral.com/1471-2458/11/455/prepub
